# Prospective insights into tibial stems, cemented versus uncemented in primary total knee arthroplasty: A dual‐energy X‐ray absorptiometry study

**DOI:** 10.1002/ksa.12758

**Published:** 2025-07-02

**Authors:** Rudy Sangaletti, Alberto Polizzi, Giacomo Capece, Luca Andriollo, Claudio Bna, Francesco Benazzo, Stefano M. P. Rossi

**Affiliations:** ^1^ Unità di Chirurgia Robotica, U.O.C. Ortopedia e Traumatologia Fondazione Poliambulanza Brescia Italy; ^2^ U.O.C. Ortopedia e Traumatologia Fondazione Poliambulanza Brescia Italy; ^3^ Università Cattolica del Sacro Cuore Roma Italy; ^4^ Artificial Intelligence Center Alma Mater Europaea University Vienna Austria; ^5^ U.O. Radiologia Fondazione Poliambulanza Brescia Italy; ^6^ IUSS Istituto Universitario di Studi Superiori Pavia Italy; ^7^ Department of Life Science, Health, and Health Professions Università degli Studi “Link Campus University” Roma Italy

**Keywords:** bone mineral density (BMD), cemented/uncemented tibial stems, implant stability, periprosthetic bone remodelling, total knee arthroplasty (TKA)

## Abstract

**Purpose:**

The durability and effectiveness of total knee arthroplasty (TKA) depend on secure implant fixation and efficient bone integration. While cemented tibial components provide immediate mechanical stability, the addition of a short stem may be indicated in selected cases to enhance fixation. However, the stem does not allow for biological integration. The advantages of cementation over press‐fit fixation remain a subject of debate, particularly regarding their impact on periprosthetic bone mineral density (BMD) over time. This study aims to compare periprosthetic BMD changes between cemented and noncemented tibial stems in primary TKA.

**Methods:**

In this prospective, monocentric study, we compared periprosthetic BMD changes in 60 patients undergoing primary TKA, randomised into two groups: 30 with cemented and 30 with noncemented tibial stems. Dual‐energy X‐ray absorptiometry (DEXA) measured BMD preoperatively and at 3, 6, 12 and 24 months postoperatively across six zones: beneath the tibial tray (R1, R2), around the stem (R3, R4), at the apex (R5), and a control zone below the stem (R6). Statistical analyses included repeated measures analysis of variance and independent *t*‐tests.

**Results:**

Cemented stems showed a significant increase in BMD at 12 months (+0.10 g/cm², *p* = 0.03) and 24 months (+0.12 g/cm², *p* = 0.04), outperforming noncemented stems (+0.06 g/cm², *p* = 0.12). Overall, cemented stems demonstrated greater BMD gains (+0.15 g/cm² vs. +0.08 g/cm², *p* = 0.03). Minimal changes were observed in both groups, with cemented stems showing slightly higher BMD retention (+0.05 g/cm² vs. −0.02 g/cm², *p* = 0.09). No statistically significant differences were recorded in certain regions.

**Conclusion:**

Cemented stems demonstrated greater periprosthetic BMD retention, which may contribute to improved mechanical environment for the implant. These findings may help guide the selection of fixation methods in primary TKA, particularly for patients with compromised bone quality.

**Level of Evidence:**

Level I.

**Abbreviations**
BMD: bone mineral density
DEXA: dual‐energy X‐ray absorptiometry
TKA: total knee arthroplasty

## INTRODUCTION

The long‐term success of total knee arthroplasty (TKA) depends on multiple factors, including proper implant alignment and fixation [13]. Tibial stems are frequently used to enhance implant stability, particularly in patients with compromised metaphyseal bone quality or high functional demands [6].

With the development of noncemented tibial plates, the option to cement or not to cement short stems has remained available. However, the stem relies entirely on press‐fit for mechanical stability and lacks of biological integration potential [5, 8].

The impact of cementation on periprosthetic bone mineral density (BMD) remains a topic of debate. While cemented implants provide immediate fixation, their long‐term effect on bone remodelling is unclear. In contrast, noncemented implants eliminate the need for cement but may struggle with early stability in patients with poor bone quality. Understanding BMD changes between cemented and noncemented tibial stems is critical, as BMD influences implant stability, micromotion and the risk of loosening [1].

The importance of cementing techniques in optimising tibial component fixation has been well recognised in the literature as described by Cawley et al. [4]. Furthermore, Driesman et al. demonstrated the advantages of cemented stems in revision TKA, particularly in cases involving poor bone quality  [5].

This study aims to compare periprosthetic BMD changes between cemented and noncemented tibial stems in primary TKA. Additionally, we seek to clarify how fixation techniques affect bone remodelling over time by measuring BMD in key anatomical regions, including beneath the tibial tray, around the stem, and at the apex. We hypothesise that cemented stems will lead to greater increases in BMD in load‐bearing areas, enhancing implant stability. Conversely, noncemented stems may promote more gradual but potentially more physiological bone adaptation. The findings of this study could have important clinical implications, guiding therapeutic decision‐making, particularly in patients with varying bone quality or a higher risk of implant failure.

## MATERIALS AND METHODS

A prospective, randomised study was conducted at the high‐volume arthroplasty centre, starting in September 2022, with a minimum follow‐up of 24 months. The study included 60 patients undergoing primary TKA, who were randomised into two equal groups: 30 received cemented tibial stems, and 30 received noncemented stems. Randomisation ensured that selection bias was minimised. All surgeries were performed by a two experienced surgeon.

Selection criteria ensured both groups were comparable in terms of demographics, clinical indications and preoperative bone health. Informed consent was obtained from all patients regarding the processing of personal data for scientific research, as well as for the planned surgical procedure. Baseline characteristics, including age, sex and preoperative BMD, were recorded. Inclusion: primary osteoarthritis, age 50–80 years, and good general health. Patients with a history of metabolic bone disorders, prior knee surgeries, or conditions affecting systemic bone metabolism were excluded. Demographics for the two groups are summarised in Table 1.

**Table 1:** Patients demographics.

Variable	Cemented (*n* = 30)	Noncemented (*n* = 30)	*p*‐Value
Age (mean ± SD)	68.4 ± 5.6 years	67.9 ± 5.8 years	0.65
Body mass index (kg/m²)	27.2	27.9	0.71
Male (%)	50%	48%	0.72
Preoperative bone density (R1–R6)	1.24 ± 0.12 g/cm²	1.22 ± 0.13 g/cm²	0.58

All procedures were performed in a high‐volume knee arthroplasty centre using the Persona® PS knee system and the ROSA robotic platform [15]. All patients received the same tibial component design, specifically the Persona® tibial tray (Zimmer Biomet) combined with a short stem extension (stubby stem), in accordance with the study protocol. The use of the stem extension was standardised across all cases and was not subject to intraoperative surgeon discretion. This approach was chosen to ensure consistency in comparing cemented and uncemented fixation strategies.

Tibial canal preparation was performed in all patients using the dedicated drill bit provided in the surgical technique guide. A retrodrill technique was employed in order to preserve and compact the trabecular bone while minimising bone removal.

**Figure 1:**
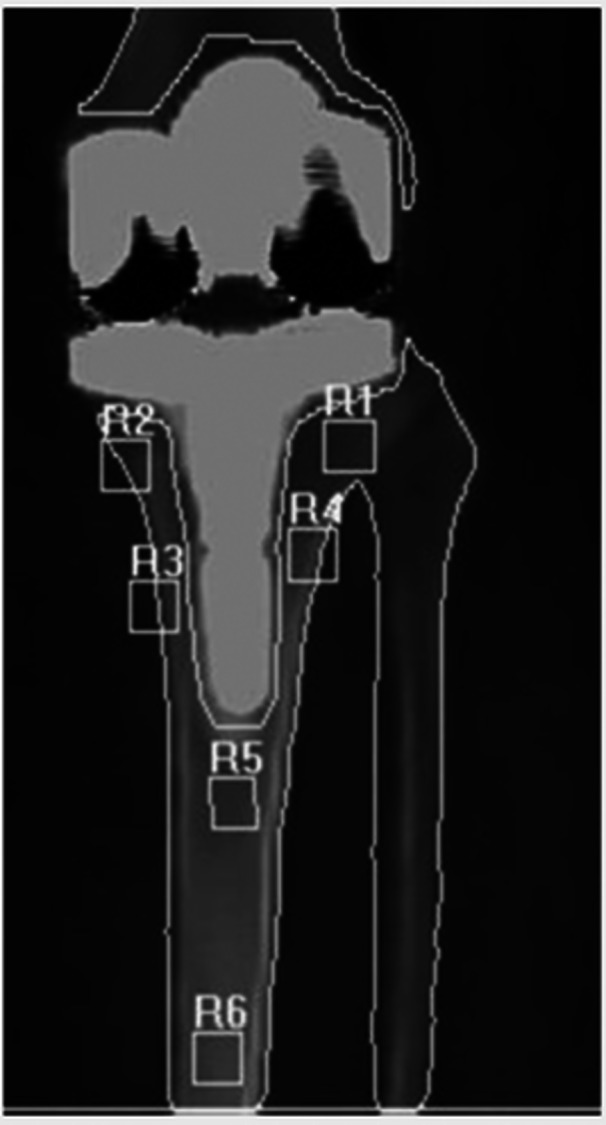
Illustration of the six anatomical zones used to assess periprosthetic bone mineral density (BMD).

BMD was assessed using dual‐energy X‐ray absorptiometry (DEXA), a validated method for quantifying bone density. Measurements were taken preoperatively (t0) and postoperatively at 3, 6, 12 and 24 months. Six anatomical zones (Figure 1) were defined to evaluate periprosthetic BMD changes [10, 12]:

- *Zones R1 and R2*: Beneath the tibial tray, representing the primary load‐bearing area.
- *Zones R3 and R4*: Around the tibial stem, where mechanical forces are transferred.
- *Zone R5*: Apex of the stem, reflecting distal fixation.
- *Zone R6*: A control region below the stem, unaffected by implant stress.

To ensure consistency, all scans were performed by the same technician, and results were reviewed by two independent radiologists blinded to group allocation.

Patients were randomised in a 1:1 ratio to receive either a cemented or an uncemented tibial stem. Randomisation was performed using a computer‐generated sequence with permuted blocks of four to ensure balanced allocation. The randomisation sequence was managed by an independent study coordinator who was not involved in patient recruitment, surgical procedures, or postoperative care, thereby minimising allocation bias. Group assignments were concealed until the time of surgery.

Ethical approval was granted by the Institutional Review Board. (NP_5035 ‘Studio CVC’). All patients provided written informed consent prior to participation, including consent for the use of their radiographic data for research purposes. The study adhered to the principles outlined in the Declaration of Helsinki.

### Statistical analysis

Descriptive statistics were used to compare demographic and clinical characteristics between groups. Repeated measures analysis of variance (ANOVA) was conducted to evaluate BMD changes over time and between groups (cemented vs. noncemented). When significant interactions between time and group were detected, post hoc comparisons with Bonferroni adjustments were performed to correct for multiple comparisons. Independent *t*‐tests were used to assess differences in BMD between the two groups at each time point (t0, 3, 6, 12 and 24 months). A significance level of *p* < 0.05 was considered statistically significant. Statistical analyses were performed using SPSS software, and graphical representations were created using GraphPad Prism.

## RESULTS

**Figure 2:**
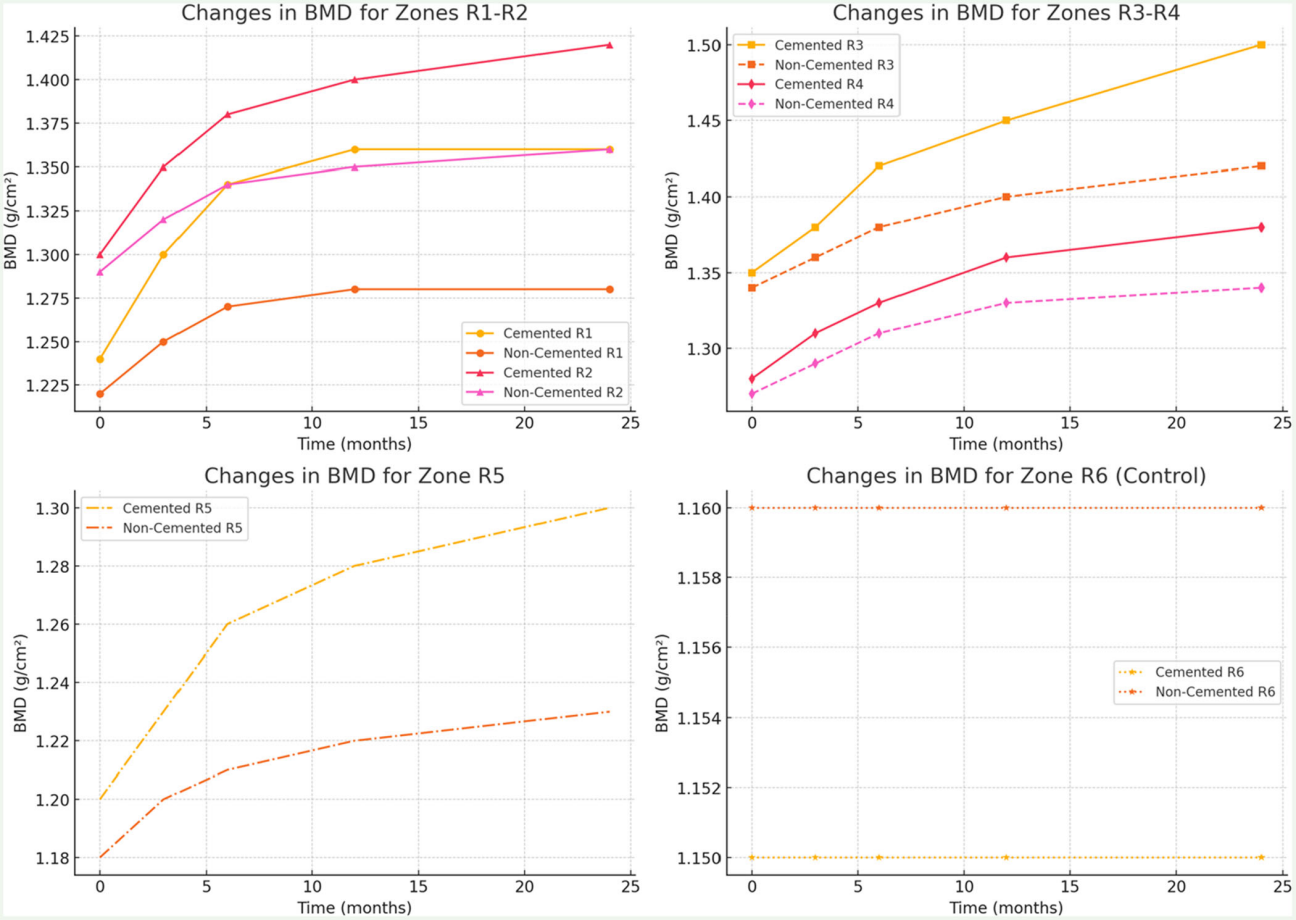
Changes in BMD. R1–R2 (under the tibial tray): A more pronounced increase in the cemented group compared to the noncemented group. R3‐R4 (around the stem): The cemented group shows a significant increase in BMD compared to the noncemented group. R5 (apex of the stem): A smaller increase compared to other zones, with differences between cemented and noncemented groups. R6 (control zone): As expected, there are no significant changes in this zone. BMD, bone mineral density.

**Figure 3:**
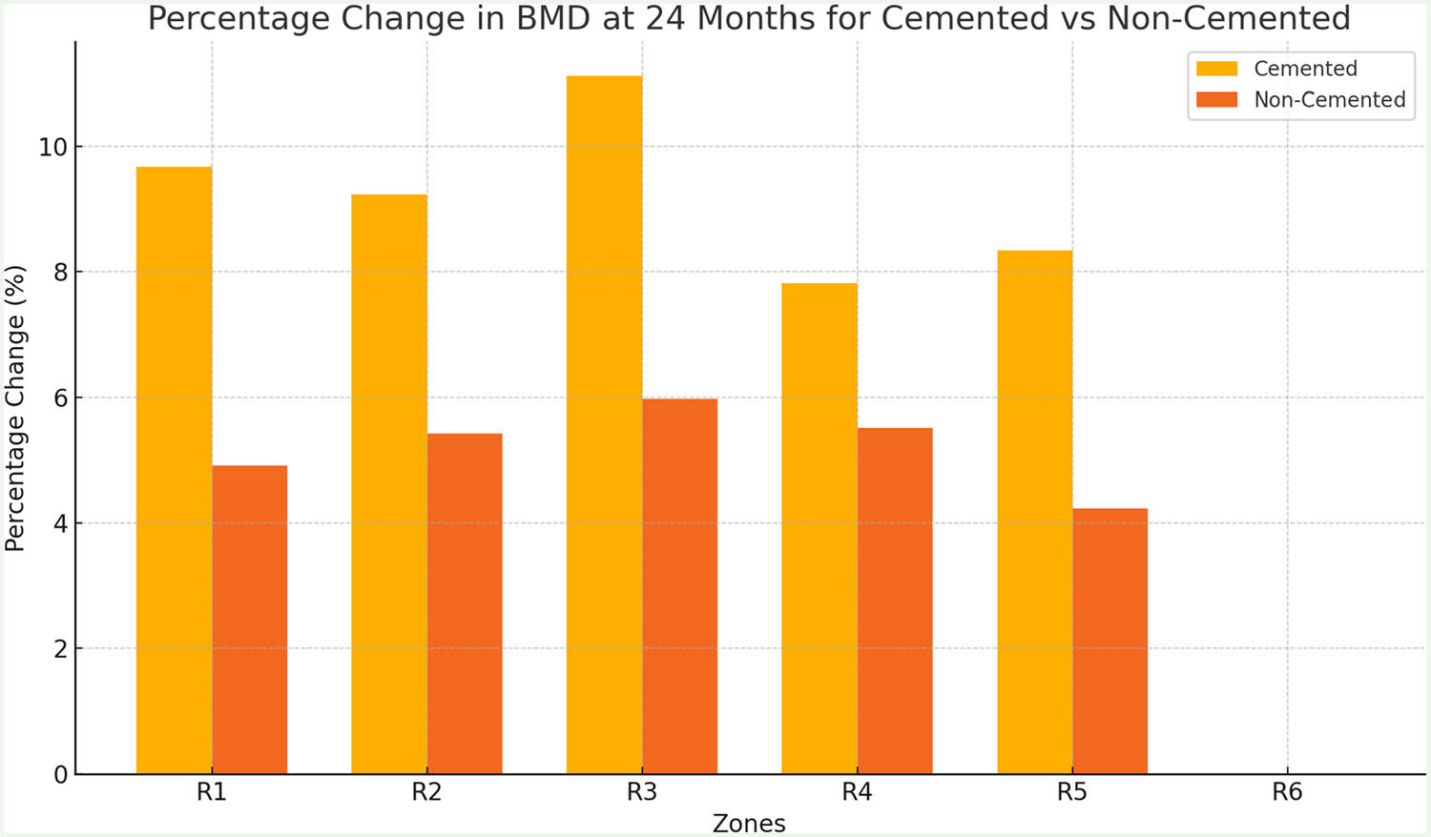
Bar chart for percentage change in BMD: This shows the percentage change in BMD across different zones (R1–R6) at 24 months, comparing the cemented and noncemented groups. The cemented group shows a greater increase in almost all areas. BMD, bone mineral density.

**Figure 4:**
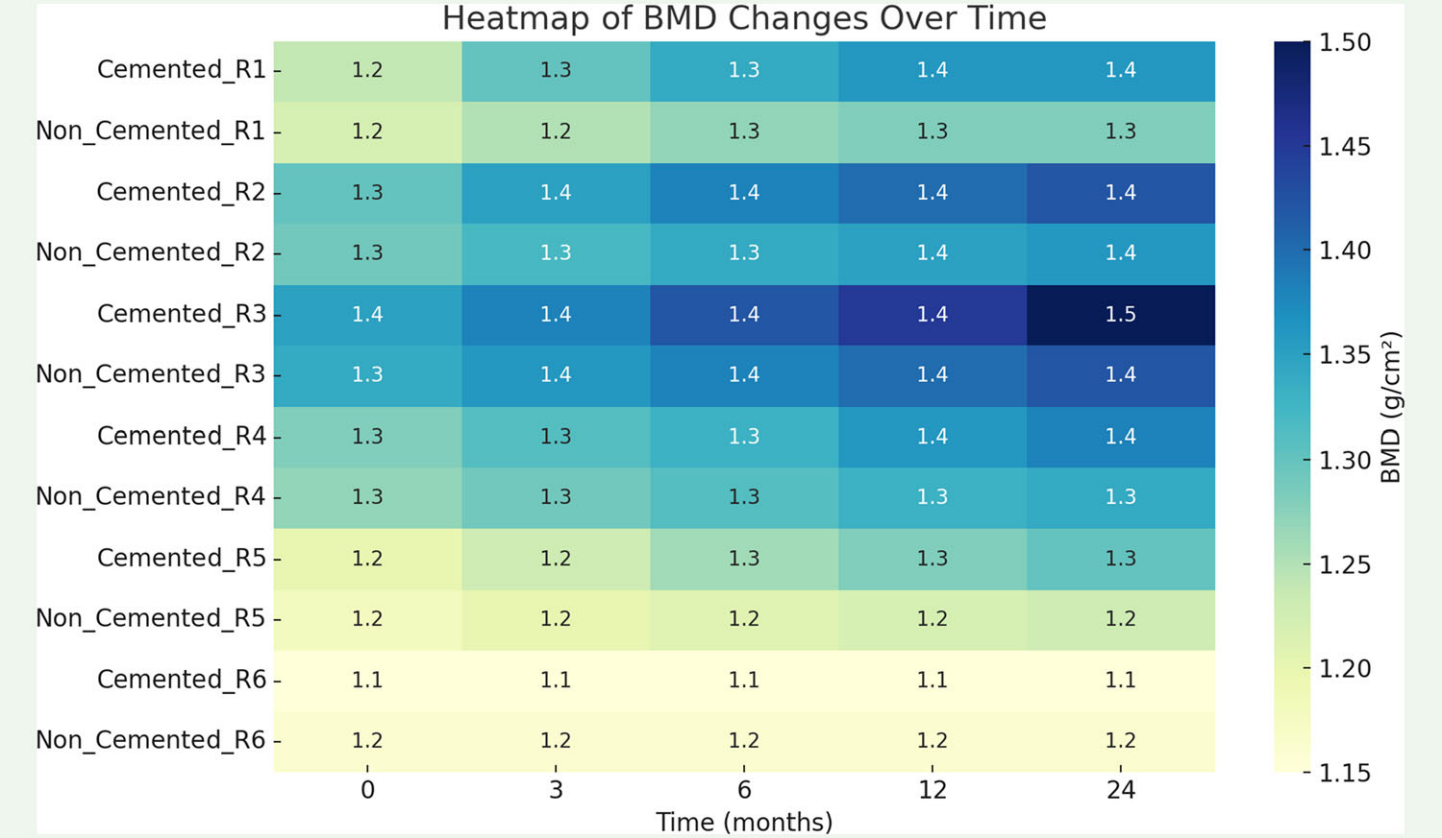
Heatmap of BMD changes over time: The heatmap displays the trend of BMD changes in different zones for both groups over time. The zones around the stem (R3–R4) in the cemented group show the highest increase in BMD. BMD, bone mineral density.

The study demonstrated clear distinctions between cemented and noncemented tibial stems in total knee arthroplasty (TKA) regarding periprosthetic BMD changes. Cemented tibial stems consistently exhibited greater increases in BMD across all load‐bearing regions, particularly beneath the tibial tray (zones R1–R2) and around the stem (zones R3–R4), compared to noncemented stems. These findings highlight the superior mechanical stability and load transfer provided by cementation (Figures 2, 3, 4, Tables 2 and 3).

**Table 2:** Baseline characteristics and preoperative bone mineral density for cemented and noncemented groups.

Zone	Cemented (mean ± SD)	Noncemented (mean ± SD)	*p*‐Value
R1	1.24 ± 0.12 g/cm²	1.22 ± 0.13 g/cm²	0.58
R2	1.30 ± 0.14 g/cm²	1.29 ± 0.15 g/cm²	0.64
R3	1.35 ± 0.16 g/cm²	1.34 ± 0.15 g/cm²	0.70
R4	1.28 ± 0.11 g/cm²	1.27 ± 0.12 g/cm²	0.66
R5	1.20 ± 0.10 g/cm²	1.18 ± 0.11 g/cm²	0.59
R6	1.15 ± 0.08 g/cm²	1.16 ± 0.09 g/cm²	0.75

**Table 3:** Periprosthetic bone mineral density changes (Δg/cm²) at 24 months in cemented versus noncemented groups.

Zone	Cemented (mean ± SD)	Noncemented (mean ± SD)	*p*‐Value
R1–R2 (24 months)	+0.12 ± 0.02 g/cm²	+0.06 ± 0.01 g/cm²	0.04
R3–R4 (24 months)	+0.15 ± 0.03 g/cm²	+0.08 ± 0.02 g/cm²	0.03
R5 (24 months)	+0.05 ± 0.01 g/cm²	−0.02 ± 0.01 g/cm²	0.09
R6 (control zone)	No change	No change	N/A

In zones R1 and R2, the cemented group showed a significant BMD increase, rising from 1.235 to 1.395 g/cm² (R1) and from 1.280 to 1.410 g/cm² (R2) over 24 months. In contrast, the noncemented group exhibited more modest increases, reaching 1.315 (R1) and 1.345 g/cm² (R2) at the same time point. The differences between the groups at 24 months were statistically significant (*p* = 0.04).

Similarly, in zones R3 and R4, the cemented group displayed significant BMD gains, increasing from 1.345 to 1.490 g/cm² (R3) and from 1.295 to 1.455 g/cm² (R4) at 24 months. By comparison, the noncemented group exhibited smaller increases, reaching 1.405 (R3) and 1.355 g/cm² (R4), with differences favouring cementation (*p* = 0.03).

At the apex of the stem (zone R5), the cemented group showed a moderate BMD increase from 1.200 to 1.285 g/cm² over 24 months, whereas the noncemented group experienced a slight decline from 1.210 to 1.195 g/cm². Although the differences were not statistically significant (*p* = 0.09), the trend suggested greater stability with cemented stems. The control zone (R6) remained stable in both groups, confirming the specificity of the observed changes.

## DISCUSSION

These findings underscore the advantages of cemented tibial stems in achieving early and robust implant fixation, particularly in patients with compromised bone quality. While noncemented stems demonstrated modest improvements, their reliance on biological fixation appeared less effective in maintaining BMD in load‐bearing zones. Cemented stems may enhance implant stability by reducing micromotion and potentially mitigating the risk of early loosening. Further studies are needed to correlate these radiographic findings with long‐term clinical outcomes, including implant longevity, functional performance and revision rates.

TKA is a well‐established and highly effective treatment for patients with severe osteoarthritis or joint degeneration, significantly improving function and reducing pain. One of the primary objectives in knee replacement is to achieve stable implant fixation to ensure long‐term durability. This study aimed to identify differences in periprosthetic BMD changes between cemented and noncemented tibial stems in primary TKA.

The results provide valuable insights into the impact of fixation methods on periprosthetic BMD.

These findings align with previous research emphasising the role of fixation in optimising implant stability and reducing the risk of early loosening.

The significant increases in BMD observed in the cemented group, especially in load‐bearing regions beneath the tibial tray and around the stem, underscore the benefits of cemented fixation in ensuring immediate mechanical stability and optimal load transfer. This mechanical advantage likely contributes to enhanced early postoperative fixation, reducing micromotion and promoting bone preservation. These findings align with the literature, reinforcing the established role of cementing techniques in primary TKA. Notably, Cawley et al. emphasised the critical importance of cementing strategies to achieve reliable and durable tibial component fixation in primary procedures  [4]. In a similar context, Driesman et al. demonstrated that cemented stems yielded superior clinical and radiographic outcomes in revision TKA, particularly in patients with compromised bone stock, where immediate fixation becomes even more pivotal  [5]. Collectively, these observations support the notion that cemented fixation remains a valuable option, especially in scenarios where bone quality and early stability are of concern.

In contrast, noncemented stems rely on biological fixation, which is influenced by bone quality and osseointegration. While the noncemented group in this study demonstrated modest BMD gains over time, the results reflect the challenges of achieving early stability in patients with lower bone density. This aligns with the findings of Andersen et al., who reported a correlation between low preoperative BMD and increased migration of uncemented tibial components [1]. Likewise, Koppens et al. highlighted the impact of bone quality on tibial component migration in cemented unicompartmental knee arthroplasty, emphasising the importance of assessing bone density when selecting fixation methods [11].

Interestingly, BMD changes at the apex of the stem (R5) were minimal, with no significant differences between the cemented and noncemented groups. This may reflect the reduced biomechanical stress in this region, as suggested by Soininvaara et al., who observed limited BMD changes in the proximal tibia following TKA [16].

Furthermore, the systematic review by Heidari et al. focused on stem extensions versus standard trays in primary cemented TKA, but did not specifically investigate the cementation of the stem itself. To our knowledge, there is limited literature directly comparing cemented versus uncemented short tibial stems in primary TKA, making the present study particularly relevant in this context. Our findings add to this sparse body of evidence by suggesting that cementing the stem may provide greater BMD retention in the short to medium term [7].

While modern cemented TKA designs have demonstrated improved outcomes, they are not without limitations. Staats et al. reported a higher incidence of radiolucent lines in modern cemented TKA designs compared to earlier models, raising concerns about potential long‐term consequences despite the benefits of immediate fixation [17]. However, the durability of cemented implants in high‐risk populations—such as obese patients—has been well documented. Fournier et al. demonstrated the effectiveness of cemented implants in ensuring implant survival in this patient group [6]. These findings are consistent with previous research, such as the study by Nikolaj et al., which demonstrated that uncemented TKA can lead to a decrease in proximal tibial BMD over time, especially in the medial region. This contrast highlights the potential benefits of cemented fixation in preserving periprosthetic bone density and ensuring implant stability in primary TKA [13].

Our findings are in line with Jaroma et al., who reported that mechanical axis correction during TKA leads to favourable bone remodelling beneath the tibial tray. Similarly, the superior BMD retention observed with cemented stems in our study highlights the role of fixation technique in promoting periprosthetic bone preservation and stability [8].

The influence of stem design and fixation methods on bone remodelling has broader implications for implant longevity and revision strategies. Kang et al. highlighted the importance of tailoring stem fixation techniques to patient‐specific factors, including bone quality and functional demands [8]. Furthermore, Jensen et al. demonstrated that porous tantalum cones in revision TKA can help mitigate BMD loss in the proximal tibia, reinforcing the role of advanced materials in supporting biological fixation [9, 14].

Several limitations must be acknowledged when interpreting these findings. First, the cohort size was relatively small, partly due to the impact of the COVID‐19 pandemic on patient recruitment and assessment. Additionally, this was a monocentric study, and data analysis was limited to information available within hospital registries.

Second, only one type of implant was used, and a robotic technique was utilised for all procedures. On the other hand, the Persona keel design was intentionally developed to optimise the best‐quality bone in the proximal tibia through its progressive medialization with size increasing. The initial design (not any more available), featuring a highly medialized keel and no stems, demonstrated promising clinical and radiological success [2, 3]. Therefore, evaluating the use of a widely adopted configuration of less medialized keel with a stem was of significant interest.

## CONCLUSIONS

Cemented tibial stems showed greater periprosthetic BMD retention over 24 months. These findings may support the use of cemented stems when early mechanical support is desired, while further studies should investigate long‐term clinical implications.

## AUTHOR CONTRIBUTIONS

Rudy Sangaletti designed and with Stefano M. P. Rossi was responsible for the study. Alberto Polizzi and Luca Andriollo contributed to write the manuscript and Rudy Sangaletti finalised the statistical section. Rudy Sangaletti and Giacomo Capece were responsible for the X‐rays evaluation. Stefano M. P. Rossi, Claudio Bna and Francesco Benazzo supervised the study and revised the manuscript.

## CONFLICT OF INTEREST STATEMENT

The authors declare no conflicts of interest.

## ETHICS STATEMENT

Ethical approval was granted by the Institutional Review Board. (NP_5035 ‘Studio CVC’). All patients provided written informed consent prior to participation, including consent for the use of their radiographic data for research purposes. The study adhered to the principles outlined in the Declaration of Helsinki.

## Data Availability

Data are available in a separate data repository and will be disclosed upon adequate request.
